# Dynamic Mechanical Properties and Damage Morphology Analysis of Concrete with Different Aggregates Based on FDM-DEM Coupling

**DOI:** 10.3390/ma17235804

**Published:** 2024-11-27

**Authors:** Kaixuan Liu, Zhenfu Chen, Qiuwang Tao, Dan Wu, Qiongfang Wu, Pinyu Zou, Minghui Wang, Yangzi Li

**Affiliations:** 1 School of Civil Engineering, University of South China, Hengyang 421001, China; 20222009110626@stu.usc.edu.cn (K.L.); chenzhenfu@usc.edu.cn (Z.C.); 2017000067@usc.edu.cn (D.W.); 2009000110@usc.edu.cn (Q.W.); nhzpy1987@sina.com (P.Z.); 20212009110531@stu.usc.edu.cn (M.W.); 20212009210563@stu.usc.edu.cn (Y.L.); 2 China Nuclear Industry Key Laboratory of High-Performance Concrete, University of South China, Hengyang 421001, China; 3 Hunan Provincial Key Laboratory of High-Performance Special Concrete, University of South China, Hengyang 421001, China

**Keywords:** FDM-DEM coupling model, microscopic parameters calibration, impact load, strain rate, different aggregates, damage pattern

## Abstract

To study the dynamic compressive mechanical properties of concrete with different aggregates (limonite and lead-zinc ore), a dynamic mechanical experiment was carried out by the Φ 100 mm SHPB equipment. Based on the coupling of the finite difference method (FDM) and the discrete element method (DEM), a three-dimensional numerical model was constructed. The effects of various strain rates and aggregate types on the dynamic mechanical properties of concrete, the dynamic increase factor (DIF), and the dynamic impact damage process were analyzed and discussed. The results show that both types of concrete have a significant strain rate strengthening effect. The dynamic compressive strength, peak strain, and DIF of the two types of concrete gradually increase with the increasing strain rate. The DIF and dynamic compressive strength growth of lead-zinc ore concrete was greater than that of limonite concrete, and the strain rate sensitivity of lead-zinc ore concrete was stronger than that of limonite concrete. The constructed three-dimensional coupling model can better simulate the experimental process, and the stress-strain curves and damage patterns show good agreement with the experimental results. The relative errors between the calibration results of the microscopic parameters and the experiment values are all within 1%.

## 1. Introduction

During its service life, concrete structures can be subjected to explosion, blasting, projectile impact, and other dynamic loading in addition to static loading [1]; their mechanical behavior under dynamic impact and quasistatic conditions differ significantly [2], so the study of their mechanical properties has been extended to dynamic mechanical properties.

The split Hopkinson pressure bar (SHPB) test system is the main way to study the dynamic mechanical properties and fracture mechanisms of materials at higher strain rates, and many scholars have utilized this test system to study the dynamic mechanical properties of different materials [3,4,5,6,7,8]. In previous research [9,10], the effect of the volume fraction of fibers on the dynamic mechanical properties of concrete was explored, and it was found that the impact resistance of concrete increased with the increase in the volume fraction of fibers, and its dynamic compressive strength also increased with the increase in strain rate. While utilizing the SHPB device to study the dynamic mechanical properties of different kinds of concrete specimens, researchers have also discovered the strain rate sensitivity possessed by concrete materials [11,12,13,14,15]; the dynamic strength of concrete increases with the increase of strain rate, and it has been widely accepted that concrete is a strain rate sensitive material, which promotes the further development of the study of the dynamic mechanical properties of concrete [16]. Zhao et al. [17] investigated the dynamic compressive mechanical properties of the mortar-concrete joint interface, and the results showed that the dynamic compressive strength of the specimens under dynamic loading increased with the strain rate, and the concrete had a stronger sensitivity to the strain rate, while the mortar had the weakest sensitivity. Yang et al. [18] explored the dynamic compressive mechanical properties of 3D printed Ultra-High-Performance Fiber-Reinforced Concrete using the SHPB test system in the mechanical properties under impact loading and observed that the dynamic increase factor (DIF) of the 3D printed specimens was anisotropic, and the specimens exhibited the most significant strain rate sensitivity in the X direction. However, researchers have only studied the dynamic mechanical properties and strain rate sensitivity of single aggregate concrete [4,19,20,21,22] without comparing different aggregate concretes; for the application of concrete in different scenarios, it is necessary to select the concrete with relatively good performance to ensure its safety, so it is valuable to compare the performance of different aggregate concretes.

While conducting the tests, numerical simulation methods have been adopted by many scholars, which is not only a common method to study the dynamic damage of concrete at the fine scale but also an effective approach to studying the dynamic mechanical properties and fracture mechanism of the material. Various numerical analysis methods have been used to simulate SHPB dynamic impact tests to characterize the mechanical properties of concrete under dynamic impact loading [23], such as the finite element software ABAQUS (https://www.3ds.com/products/simulia/abaqus) [24,25,26], ANSYS (https://www.ansys.com/zh-cn) [27], and the discrete element software PFC (https://www.itasca.cc/cpzx) [28,29,30]. Liu et al. [31] studied the interface effect between specimen and rod in the SHPB test and carried out corresponding numerical simulation with ABAQUS, analyzed the sensitivity of the interfacial friction coefficient, specimen diameter, and specimen unevenness, and derived corresponding sensitivity ranking, which provided an effective method to improve the accuracy of the test. Along with the further depth of the research, the mechanical law of concrete must be studied from the fine-scale level, therefore, the advantage of the discrete element method is gradually expanding, which overcomes the limitations of the traditional macro continuity assumption and has been adopted by many scholars. Chen et al. [32] simulated the SHPB impact compression test of concrete under different strain rates by using the discrete element software PFC2D (https://www.itasca.cc/cpzx) to establish the fine-scale granular cell model, and the results show that the simulated stress-strain relationship and dynamic strength improvement law of concrete are consistent with the test results, which verifies the feasibility of the simulation. Wang et al. [33] used the discrete element software PFC3D (https://www.itasca.cc/cpzx) to carry out numerical simulation experiments on the damage process of coal bodies under different impact velocities and explored the influence of impact velocity, fissure distribution angle, and other factors on the damage characteristics of coal body, and the results show that when the impact velocity is lower, the coal samples mainly follow the deformation damage criterion; additionally, under the action of the uniaxial impact load, the coal samples are mainly damaged by the form of tensile damage, and the simulation results can be corroborated with the experiments, reflecting the strain rate effect. In terms of discrete meta-studies, researchers have all used only a single 2D or 3D model for the relevant studies and have not fully simulated SHPB experiments. Most of these simulations on SHPB tests use a single numerical simulation method (finite element method or discrete element method), in which, although the finite element method has high computational efficiency, it is difficult to effectively analyze the fine-scale aspects such as crack development and damage evolution in the material; whereas the discrete element method, although it has a great advantage in the simulation of the fine-scale aspects and the analysis of the crack development, due to the fact that the rods in the SHPB test setups are high-strength steels that only undergo minor deformations, it is not possible to effectively simulate the rods with the discrete element method to ensure the simulation accuracy. Therefore, using only a single finite element of continuous medium or a discrete element of discrete medium cannot effectively and completely simulate the SHPB test process, which has certain limitations [34,35].

In this work, the dynamic mechanical experiment was carried out by the Φ 100 mm SHPB equipment (Luoyang Livy Technology Co., Ltd., Luoyang, China). Based on the coupling of FDM and DEM, a three-dimensional numerical model that can completely simulate the SHPB experiment was established. Using the PFC6.0 built-in Python language module, a parameter autocalibration program was written to calibrate the microscopic parameters of the constructed model. The influence laws of strain rate and aggregate type on the dynamic mechanical properties of concrete, DIF, and dynamic impact damage process were investigated.

## 2. Experiment

### 2.1. Specimen Preparation and Basic Mechanical Properties

The static test was conducted in accordance with the Standard for Test Methods of Mechanical Properties of Ordinary Concrete (GBT50081-2002) [36] to prepare the required concrete specimens, and the static compressive strength test and elastic modulus determination test were carried out in strict accordance with the standard [21,22]. The basic physical properties of concrete specimens are shown in Table 1.

The SHPB impact compression test was conducted in accordance with the “Standard Test Methods for Mechanical Properties of Ordinary Concrete” (GBT50081-2002) to prepare the required specimens; the specimens are cast in molds with a diameter of 100 mm and a height of 50 mm. After standard curing for 28 d, due to the roughness of the surface of the test block, the test piece needs to be smoothed using the double-end grinding machine to make the specimen end face flatness to meet the requirements of 0.01 mm, eliminate the appearance of the specimen with obvious natural and processing defects, and to ensure the accuracy of the test results.

### 2.2. SHPB Test Results

**Figure 1:**
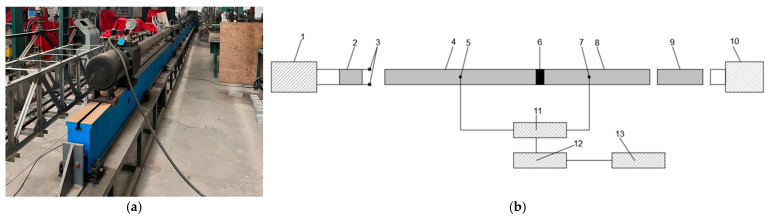
Split Hopkinson pressure bar (SHPB) equipment; (**a**) actual picture; (**b**) schematic: 1—launching device; 2—bullet; 3—velocimeter; 4—incident rod; 5—strain gauges; 6—specimen; 7—strain gauges; 8—transmissive rod; 9—absorbent rod; 10—damping device; 11—ultra-dynamic strain gauge; 12—intelligent measurement analyzer; 13—data processing system.

The SHPB device used in the test is shown in Figure 1. The device consists of three parts: the pressurized rod system (including devices such as bullets, incident rods, transmissive rods, absorber rods, and dampers), the data acquisition device (devices such as pneumatic pressure controllers, laser velocimeters, and hyperdynamic strain gauges) and the data processing system. Among them, the lengths of the bullet, the incident rod, the transmission rod, and the absorption rod are 0.6 m, 5 m, 3 m, and 2 m, respectively, and the diameters are 100 mm, all of which are made of the same kind of steel, with the elastic modulus E = 190.3 GPa, the density ρ = 7650 kg/m^3^, and the Poisson’s ratio ν = 0.3. The supports of the incident, transmission, and absorption rods are all ball bearings, which makes the rods subject to much less friction during movement and, at the same time, more convenient to use. The strain gauge resistance value is 120 + 0.2% Ω, and the sensitivity factor is 2.12 + 1.3%. The test acquisition system is the DH5960 Ultra-Dynamic Signal Test and Analysis System, which was used to amplify the strain signal for better analysis and processing of data.

In order to minimize the friction effect on the test results, the interface between the rod and the test piece is fully coated with lithium grease. During the SHPB test, the specimen is placed between the incident and transmitted rods, and the impact velocity of the bullet is controlled by adjusting different air pressure values, which in turn controls different strain rates. Strain gauges are pasted on the incident and transmitted rods to record the changes in the waveform signals of the incident, as well as reflected and transmitted waves during the test. The average value is taken when calculating, which is conducive to reducing the error. The propagation speed of the incident wave is determined by the modulus of elasticity and density of the incident rod, and the propagation time is determined by the length of the incident rod and the wave speed. The measured data can be calculated and processed to derive the stress-strain curve of the specimen material under dynamic loading to reflect the various dynamic properties of the material.

The dynamic load calculation in the SHPB test is mainly based on the one-dimensional elastic wave assumption and stress uniformity assumption. In this paper, the “two-wave method” is adopted to calculate the stress $\sigma \left(t\right)$, strain rate $\overset{•}{\varepsilon }\left(t\right)$, and strain $\varepsilon \left(t\right)$ of concrete specimens. The calculation formula is shown in Equation (1) [37].

(1) $$\left\{\begin{array}{c} \sigma \left(t\right)=\frac{EA_{0}}{A_{s}}\varepsilon_{1}\left(t\right)\\ \overset{•}{\varepsilon }\left(t\right)=-\frac{2C_{0}}{l_{s}}\varepsilon_{r}\left(t\right)\\ \varepsilon \left(t\right)=-\frac{2C_{0}}{l_{s}}\int_{0}^{t}\varepsilon_{r}\left(t\right)dt \end{array}\right.$$

where $\sigma \left(t\right)$ is the stress inside the specimen at moment *t* during the impact process; $\overset{•}{\varepsilon }\left(t\right)$ is the strain rate inside the specimen at moment t during the impact process; $\varepsilon \left(t\right)$ is the strain inside the specimen at moment t during the impact process; t is the time; E is the modulus of elasticity of the incident and transmitted rods; $A_{0}$ is the cross-sectional area of the incident and transmitted rods; $A_{s}$ is the cross-sectional area of the specimen; $C_{0}$ is the propagation velocity of the stress wave in the rod; $l_{s}$ is the height of the specimen; $\varepsilon_{1}\left(t\right)$ is the strain signal of the transmitted wave in the compression rod; $\varepsilon_{r}\left(t\right)$ is the strain signal of the reflected wave in the compression rod.

**Figure 2:**
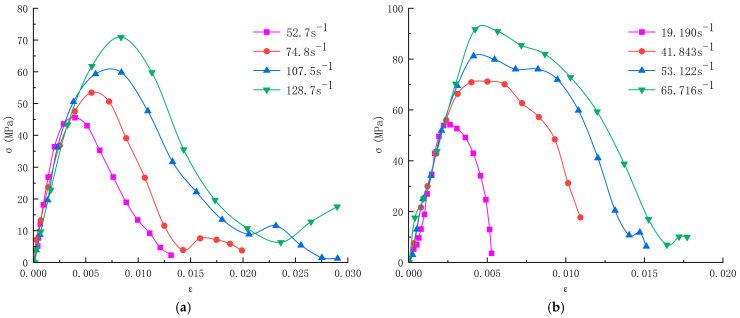
Stress-strain curves of concrete with different aggregates at different strain rates; (**a**) limonite concrete; (**b**) lead-zinc ore concrete.

The data collected by the collection system were calculated using Equation (1) to obtain the stress-strain curves of limonite concrete and lead-zinc concrete at different strain rates, as shown in Figure 2.

## 3. Numerical Simulation

### 3.1. Numerical Modeling

In this paper, SHPB impact compression test simulations were performed based on the coupling of the discrete element method PFC3D6.0 and the finite difference method FLAC3D6.0. The numerical model of the specimen was built using the PFC3D6.0 module and was first formed into a cylindrical region formed by three walls, in which spherical particles with uniformly distributed radius ranging from 0.8 mm to 1.44 mm were later generated, with a total of 37,093 particles. The contact model between particles is a linear parallel bond model, which is capable of transmitting both force and moment and can perform the simulation of macroscopic and microscopic force characteristics and crack extension of the simulated materials. Afterward, the generated specimens were pre-compressed (simulating the state of the specimen in the mold) and then cemented, generating a total of 172,882 contacts (including particle-to-particle contact and particle-to-wall contact), followed by unloading (simulating the state of the specimen when the specimen is taken out of the mold), and the contacts were reduced to 169,331 (the reduced contacts were between the wall and the particles). The dimensions of the modeled specimens were consistent with the test dimensions. The specific parameters are shown in Table 2.

**Figure 3:**
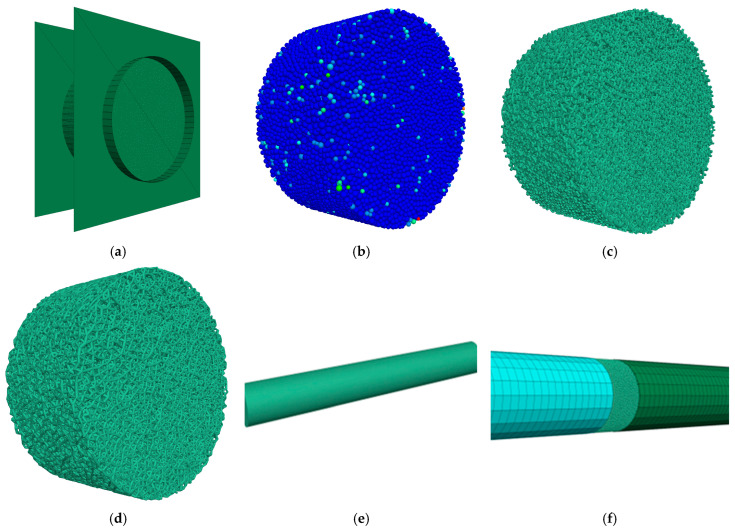
Schematic diagram of discrete element specimen numerical model establishment; (**a**) sample formation; (**b**) precompression; (**c**) cementation; (**d**) unload; (**e**) bullet; (**f**) load.

The bullet is built by using the Rigid Block module in PFC3D6.0 with a length of 600 mm; the numerical models of the incident and transmitted rods are built by using the FLAC3D6.0 module to achieve the uniform transmission of the stress wave in the SHPB test system. The length of the incident and transmitted rods is 5000 mm, and their diameters are 100 mm. The parameters of the established numerical models, including the length, diameter, and material properties of the rods, are consistent with those of the test setup, as shown in Table 3. The numerical model established based on the PFC3D6.0-FLAC3D6.0 coupling is shown in Figure 3.

In order to monitor the transmission of stress waves on the rods, monitoring points are set up on the incident rods and transmissive rods, respectively, to monitor the stress and strain changes of the rods in order to compare with the stress-strain curves of the specimens derived from the tests, a measuring sphere is set up at the center of the simulated specimens to monitor the stress and strain changes of the specimens.

As far as the coupling part is concerned, the coupling logic works by obtaining the contact forces and moments with the wall and determining the equivalent force system at the face vertex, which is transferred to the mesh points/nodes together with the stiffness contribution. The coupling between PFC3D6.0 and FLAC3D6.0 includes wall zone (face domain) coupling based on boundary control wall and ball-zone (ball domain) coupling based on boundary control particles. In this paper, wall-zone (fac domain) coupling based on boundary control wall is used to achieve the mechanical connection between the zone (rod) and ball (specimen).

### 3.2. Microscopic Parameter Calibration

Microscopic parameter calibration is the bridge between macro and micro in discrete element simulation. In the discrete element model, the macroscopic intrinsic parameters of the material cannot be directly assigned to the particles; the macroscopic mechanical properties are reflected by the microscopic parameters, so the accuracy of the values of the microscopic parameters determines the accuracy of the simulation results.

**Figure 4:**
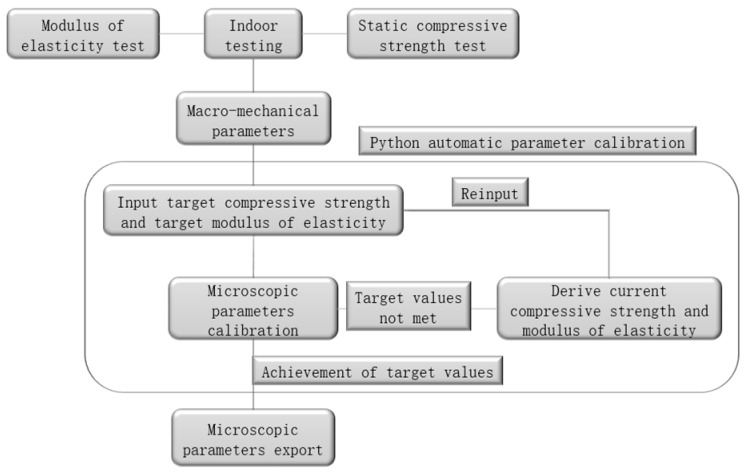
Flow chart for the calibration of concrete microscopic parameters.

**Figure 5:**
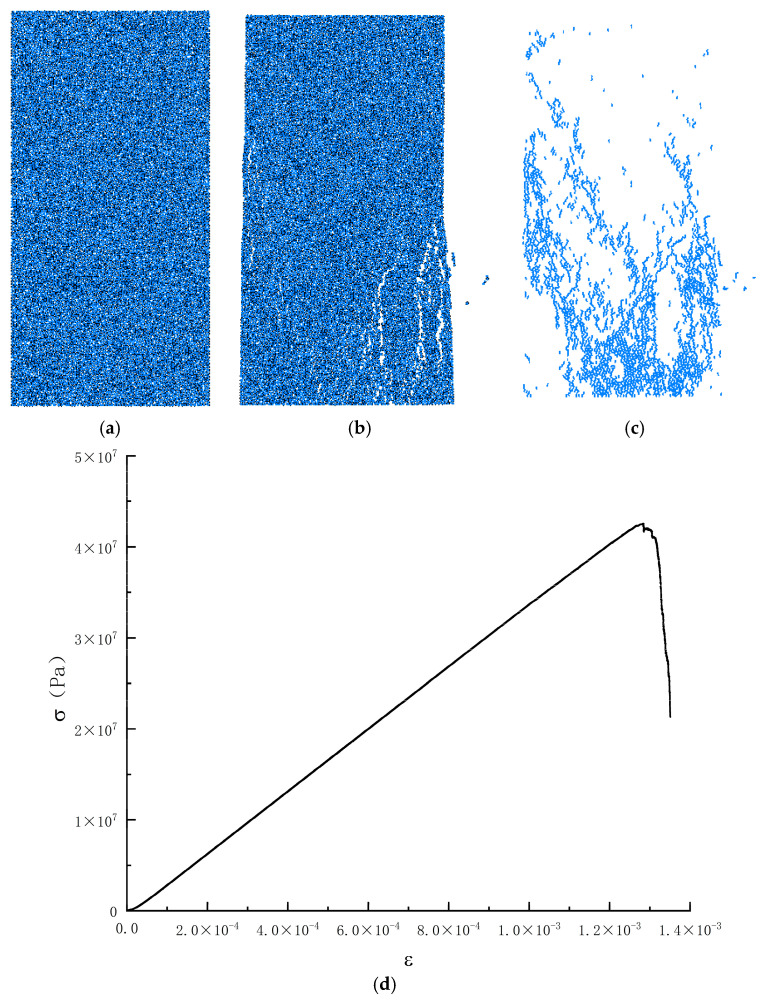
Schematic diagram of the parameter calibration of a numerical model for limonite concrete; (**a**) specimen diagram; (**b**) damage diagram; (**c**) rift diagram; (**d**) numerical simulation of uniaxial compression stress-strain curves.

**Figure 6:**
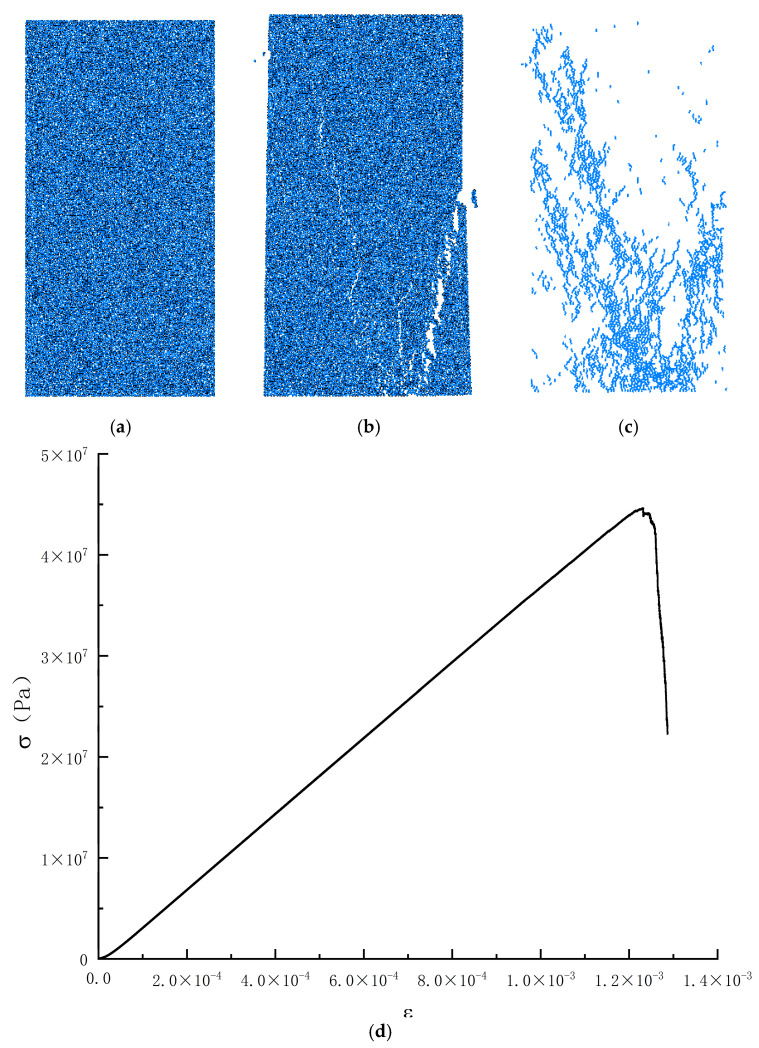
Schematic diagram of the parameter calibration of a numerical model for lead-zinc ore concrete; (**a**) specimen diagram; (**b**) damage diagram; (**c**) rift diagram; (**d**) numerical simulation of uniaxial compression stress-strain curves.

This paper is based on the discrete element software PFC2D6.0 based on the unconfined uniaxial compression test as a unit experiment for parameter calibration; the calibration process mainly adopts the built-in Python programming language of PFC6.0, based on the Python language to write the parameter calibration code, set up the target compressive strength and the target elasticity modulus, initially endowed with the initial value of certain microscopic mechanical parameters. After the program is run, through repeated comparison and correction with the set target values, the calibration of the microscopic parameters is automatically conducted to achieve the maximum matching degree, and the appropriate microscopic parameters can make the mechanical properties of the numerical model closer to the macro-mechanical properties of the test. The calibration process is shown in Figure 4. The calibration results are shown in Figure 5 and Figure 6.

In this paper, parameter calibration is carried out based on a linear parallel bonding model contact, and the interparticle forces mainly follow Newton’s second law and force-displacement law during the computational run of the discrete element particle flow software PFC6.0, independent of the deformation coordination.

The microscopic parameters of the linear-parallel bonding model mainly include particle linear contact microscopic parameters and linear-parallel bonding microscopic parameters, which need to be calibrated as follows: the particle part includes emod (effective modulus), kratio (normal-shear stiffness ratio), fric (coefficient of friction), and the bonding part includes pb_emod (bonding effective modulus), pb_coh (cohesion of bonding), pb_ten (bond tensile strength), pb_fa (bond internal friction angle), pb_kratio (bond normal-shear stiffness ratio); emod, pb_emod, kratio, and pb_kratio are deformation parameters, and fric, pb_coh, pb_ten, and pb_fa are strength parameters. The strength parameter has no effect on deformation, while the deformation parameter affects the stiffness and also has some effect on strength [38]. In this paper, we mainly control the strength by controlling pb_coh and the stiffness by controlling emod/pb_emod.

The results of the compressive strength and modulus of elasticity derived from numerical simulation are consistent with the test results (see Table 4). It is observed that the simulated macro-parameters of compressive strength and modulus of elasticity obtained for limonite concrete have an error of 0.366% and 0.036%, respectively, from the target values of the macro-parameters of indoor tests, while the simulated macro-parameters of compressive strength and modulus of elasticity obtained for lead-zinc ore concrete have an The errors are 0.486% and 0.185%, respectively, and the corresponding errors are below 1%, and this error is considered acceptable [39]. The corresponding parameters at this point are the most appropriate microscopic parameters for this paper. The simulated microscopic parameters of concrete with different aggregates are shown in Table 5 and Table 6.

## 4. Results and Analysis

### 4.1. Model Validation

In performing the SHPB numerical simulation of dynamic impact compression tests, the required simulation results are obtained by applying different impact velocities to the bullets so that the bullets impact the incidence rods in order to apply different strain rates to the specimens to simulate the damage process of concrete with different aggregates at different strain rates by means of the measurement points set up on the incidence rods, the transmissive rods, and the specimens.

**Figure 7:**
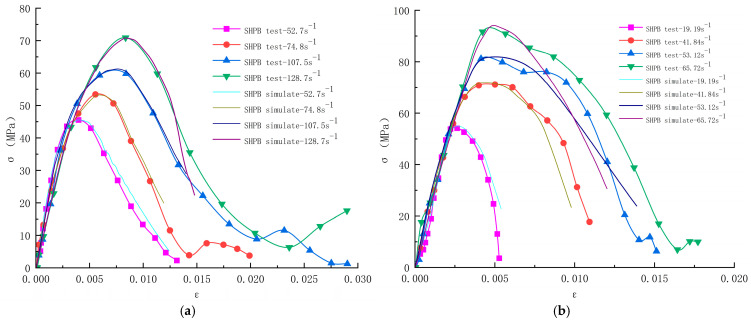
Experimental and simulated stress-strain curves of concrete with different aggregates at different strain rates; (**a**) limonite concrete; (**b**) lead-zinc ore concrete.

The comparison of the stress-strain curves obtained by numerical simulation with the SHPB test curves is shown in Figure 7, which shows that the results of numerical simulation are consistent with the SHPB test results. As the strain rate increases, the slope of the rising section of the curve becomes larger, which indicates that the deformation resistance of the specimen is improved. The slope of the rising section of each simulated curve is the same as that of the rising section of the test curve, and the peak stress of the simulated curves, as well as the strain corresponding to the peak stress, are also close to the test curves, which indicates that the practice of automatic parameter calibration to derive the microscopic parameters by Python in the simulation is feasible, and the determined microscopic parameters are reasonable.

By analyzing Figure 7, the dynamic stress-strain curve can be roughly divided into three stages:

(1) Elastic growth stage. In the initial stage, there is an approximately linear relationship between stress and strain; the stress increases rapidly with the increase of strain, and the slope under the strain rate is similar in all groups; the concrete is in the elastic deformation stage at this stage, the stress inside the specimen gradually begins to homogenize, and the deformation is mainly dependent on the deformation of the cement mortar and aggregate inside the concrete. With the gradual increase of strain rate, the slope of the initial section of the curve increases but is not obvious, indicating that the elastic modulus is unaffected by the strain rate at this stage.
(2) Elastic-plastic deformation stage. With the increasing stress, close to the peak stress point, the stress and strain no longer have a linear relationship; the concrete began to enter the elastic-plastic deformation stage, and the internal pores and cracks began to be extruded, with the continuous expansion of micro-cracks, the concrete produces an irrecoverable deformation, the slope of the curve is a nonlinear decrease in the strain rate, the higher, the greater the peak stress and the peak strain of the concrete. When the peak stress is reached, the concrete is extruded into a dense state, at which time the stress of the concrete reaches the maximum value.
(3) Plastic damage stage. When the peak stress is reached, the concrete reaches the ultimate yield strength, and new cracks are constantly generated within the concrete and rapidly extend and expand to form the damaged surface; these cracks do not have sufficient time to complete the process of re-squeezing, at this time, the stress drops rapidly, the strain continues to increase, the concrete appears to be brittle damage, and the stress-strain curve shows a rapid downward trend.

**Figure 8:**
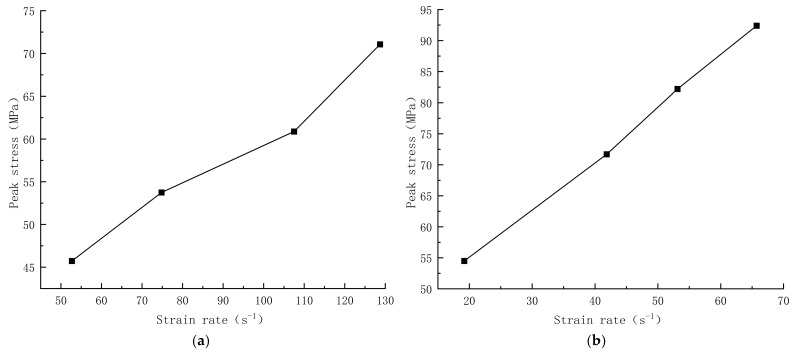
Peak stress versus strain rate for concrete with different aggregates; (**a**) limonite concrete; (**b**) lead-zinc ore concrete.

The relationship between peak stress and strain rate for concrete with different aggregates is shown in Figure 8. Figure 8 shows that the dynamic compressive strengths of both concrete exhibit strain rate dependence, and the peak stress increases with the strain rate in an approximately linear trend. The dynamic compressive strength of limonite concrete ranged from 46 to 71 MPa, which was greater than that of limonite concrete under quasistatic loading (42.4 MPa); the dynamic compressive strength of lead-zinc ore concrete ranged from 54 to 92 MPa, which was greater than that of lead-zinc ore concrete under quasistatic loading (44.4 MPa). The increase in dynamic compressive strength of lead-zinc ore concrete was greater than that of limonite concrete, and the bearing capacity of lead-zinc ore concrete for impact loading was higher than that of limonite concrete.

The stress-strain curves of limonite concrete and lead-zinc ore concrete showed similar trends, and both experienced the same stage, i.e., the stress and strain were approximately linear at the initial stage and then the slope of the curve decreased nonlinearly and decreased sharply after the stress reached the peak value, which illustrated the strain-softening phenomenon after the peak stress and the specimens appeared to be broken to different degrees. Both concretes have an obvious strain rate effect, i.e., the dynamic compressive strength gradually increases with the increase of strain rate, which is significantly different from the mechanical properties at both static loadings.

In summary, the simulation method and the three-dimensional coupling model used in this simulation are reasonable and reliable, and the resulting relevant curves match the test results to a certain extent, indicating that the coupling of the discrete element method (PFC3D6.0) and the finite difference method (FLAC3D6.0) to establish the relevant numerical model can simulate the SHPB impact compression test in a better way, and analyze effectively the concrete under impact loading.

### 4.2. Strain Rate Effect

**Figure 9:**
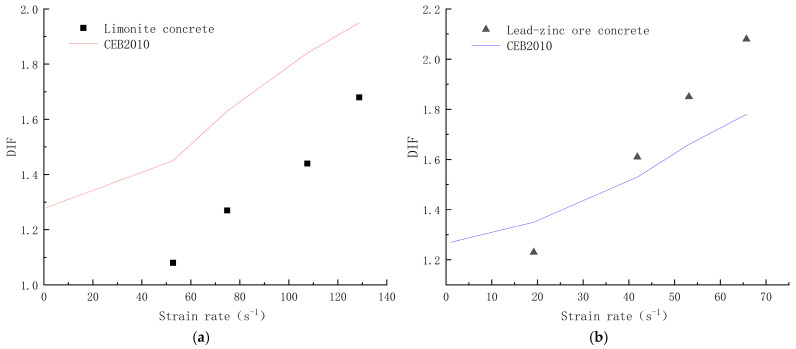
DIF vs. strain rate for limonite concrete and lead-zinc ore concrete; (**a**) limonite concrete; (**b**) lead-zinc ore concrete.

The Dynamic Increase Factor (DIF) is generally used to describe the proportion of the increase in the dynamic compressive strength of concrete under different strain rates, characterizing the change in the dynamic compressive strength of the material under dynamic loading, which is defined as the ratio of the dynamic compressive strength of concrete to quasistatic compressive strength [4]. Currently, the widely used equation is the CEB2010 equation [40]. The DIF obtained in this paper is compared with the calculation results of the CEB2010 equation, as shown in Figure 9.

The results of this paper have large differences with the curves obtained from the CEB2010 formula, indicating that the formula is not applicable in this paper. According to this analysis, on the one hand, it may be the influence of the different software, instruments, and data processing methods used, and on the other hand, it may be that the calculation formula of DIF itself has not been agreed upon, and the values of static compressive strength are different. The static compressive strength values used in this test refer to the 28 d compressive strength values of cubes.

As can be seen from Figure 9, the DIF of both concretes increases with the increase of strain rate, which indicates that both limonite concrete and lead-zinc ore concrete have strain rate sensitivity, and both of them have obvious dynamic reinforcement effect; the DIF of lead-zinc ore concrete increases more than that of limonite concrete with the increase of strain rate.

**Figure 10:**
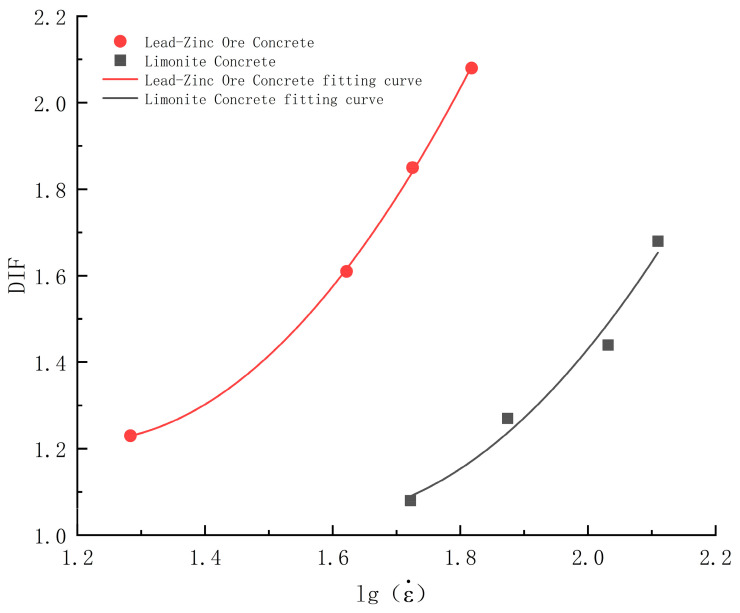
Fitted curves of DIF vs. logarithm of strain rate for limonite concrete and lead-zinc ore concrete.

In order to compare the strain rate sensitivity of limonite concrete and lead-zinc ore concrete and to obtain the relationship between DIF and strain rate for limonite concrete and lead-zinc ore concrete, the fitted relationship curves and relationship equations for the logarithm of DIF and strain rate for limonite concrete and lead-zinc ore concrete can be proposed. The fitted curves are shown in Figure 10. The fitted empirical formula and correlation are shown in Equation (2).

(2) $$\left\{\begin{array}{c} {DIF}_{\left(\mathrm{HC}\right)}=2.08{\left(lg\dot{\varepsilon }\right)}^{2}-6.53\left(lg\dot{\varepsilon }\right)+6.16,\;R^{2}=0.98\\ {DIF}_{\left(\mathrm{PC}\right)}=2.34{\left(lg\dot{\varepsilon }\right)}^{2}-5.64\left(lg\dot{\varepsilon }\right)+4.62,\;R^{2}=0.99 \end{array}\right.$$

where ${DIF}_{\left(\mathrm{HC}\right)}$ is DIF of limonite concrete; ${DIF}_{\left(\mathrm{PC}\right)}$ is DIF of lead-zinc ore concrete.

Analysis of Figure 10 and Equation (2) shows that the slope of the fitted curves for lead-zinc ore concrete is greater than that for limonite concrete, indicating that the rate correlation of lead-zinc ore concrete is stronger than that of limonite concrete.

It should be noted that Equation (2) is derived based on the data of this thesis and is applicable within certain limits.

### 4.3. Damage Patterns

**Figure 11:**
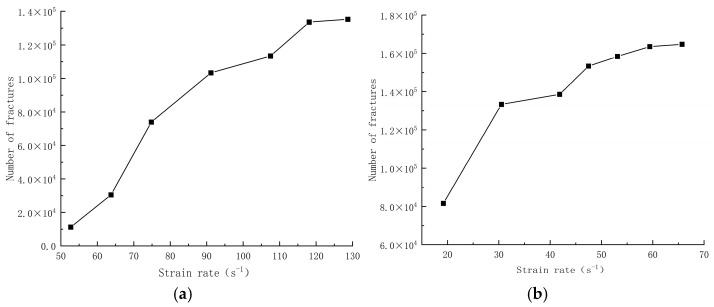
Relationship between number of cracks and strain rate in concrete with different aggregates; (**a**) limonite concrete; (**b**) lead-zinc ore concrete.

The degree of damage caused by different strain rates on concrete specimens is not the same, and the damage morphology presented is also different. The test results show that there are two kinds of damage morphology of specimens under different strain rates: block damage and powder damage. The damage patterns of limonite concrete and lead-zinc ore concrete under different strain rates are shown in Table 7 and Table 8. The relationship between the number of cracks produced and the strain rate after the damage of limonite concrete and lead-zinc ore concrete is shown in Figure 11. It should be noted that in the 3D simulated crack diagrams, the internal cracks produced by the damage of concrete under strain rate recorded by the software are disk-shaped.

By analyzing Table 7 and Table 8, it can be found that the damage pattern evolves from crushing into large pieces to crushing into small pieces as the strain rate increases. At lower strain rates, the size of broken pieces after the crushing of specimens is generally larger, and with the increase of strain rate, the size of broken pieces after the crushing of specimens decreases gradually, and the disintegration of concrete results in the splattering of fragments.

As can be seen from Figure 11, the number of cracks increases with the increase of strain rate, indicating that the degree of specimen fragmentation increases with the increase of strain rate, which coincides with the damage morphology shown in the test. Comparing the damage morphology between the test and simulation, it can be found that under the action of low strain rate, the cracks of concrete mainly expand along the mortar interior or the mortar and aggregate interface; at this time, the formation of penetration cracks is less, and the performance is a broken block is larger. With the increase of strain rate, the concrete internal production of multiple cracks was diffused to carry out, the whole damage process of tensile cracks and shear cracks randomly generated, cracks to different directions, different levels of development through the direct formation of one or several through the entire specimen of the crack, the specimen is broken out of the block is further reduced to a smaller size, and then the degree of fragmentation intensified, the entire specimen to form macroscopic cracks leading to the destruction of the concrete.

### 4.4. Crack Evolution Analysis

**Figure 12:**
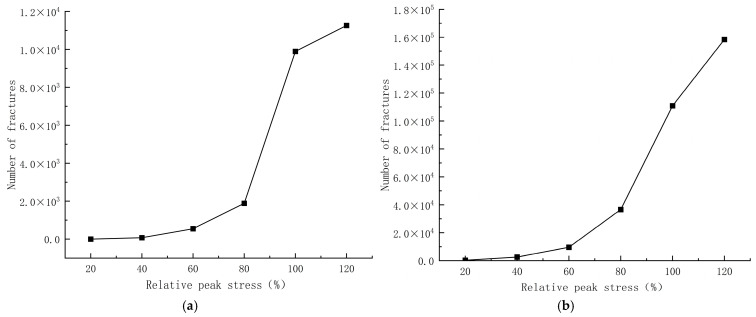
Variation in the number of cracks in concrete with different aggregates for different stress cases at similar strain rates; (**a**) limonite concrete; (**b**) lead-zinc ore concrete.

The SHPB impact compression test can analyze the dynamic mechanical properties of concrete specimens at the macroscopic level, but due to the short loading time, it is impossible to accurately observe the crack evolution process of the specimens at the fine-scale level without the aid of tools. While discrete elements can observe and analyze the crack development of specimens under different stress states from the fine level, combining tests and simulations is convenient for describing the crack evolution process of concrete more comprehensively. The crack evolution process of concrete with different aggregates under different stress conditions at similar strain rates is shown in Table 9. The evolution of the number of cracks in concrete with different aggregates for different stress cases at similar strain rates is shown in Figure 12.

Table 9 shows that for limonite concrete specimens or lead-zinc ore concrete specimens (horizontal comparison), the crack evolution can be roughly divided into three stages: (1) Crack emergence stage. In the initial stage of loading, cracks are mostly generated at the junction of the specimen and the compression bar, and are discretely distributed, small in number, and have little effect on the concrete. This corresponds to the elastic growth stage of concrete; (2) crack development stage. With further loading, the specimen is gradually compacted, and cracks begin to arise inside the specimen; with the development of time, the number of cracks increases rapidly, and there are many interconnected cracks, which will have greater damage to the concrete, but at this time, there are still some cracks in a discrete state, which does not affect the damage to the concrete. This time corresponds to the elastic-plastic stage of concrete; (3) crack development specimen destruction stage. After reaching the peak stress, the internal and external cracks expand and eventually connect with each other, forming a penetrating crack so that the specimen is broken into multiple fragments, which corresponds to the plastic phase of concrete.

Observing Figure 12 and comparing the crack evolution of limonite concrete and lead-zinc ore concrete for different stress scenarios at similar strain rates (longitudinal comparison), it can be observed that the number of cracks in lead-zinc ore concrete is much larger than that in limonite concrete at the same stress stage. With the gradual increase in stress, the number of cracks in lead-zinc ore concrete grows much faster than in limonite concrete. At 80% peak stress to 100% peak stress, the number of cracks in both limonite concrete and lead-zinc ore concrete increased at a maximum rate. It also confirms that the dynamic compressive strength increase of lead-zinc ore concrete is greater than that of limonite concrete.

### 4.5. Analysis of Contact Anisotropy and High Stress Distribution in Specimens

Histogram analysis is an important analytical tool in bulk mechanics. In this paper, the main purpose is to use the direction of the force in the particles to statistically analyze the direction of the force, which can be reflected in the contact anisotropy state of the specimen through the group configuration diagram. The way to derive the contact anisotropy law of concrete is to first traverse each contact and divide the different zones by counting the directions the contact (contact is not a vector in PFC6.0, it is a line between two particles, and the contact can be given a different direction by a concept of direction built in PFC6.0).

**Figure 13:**
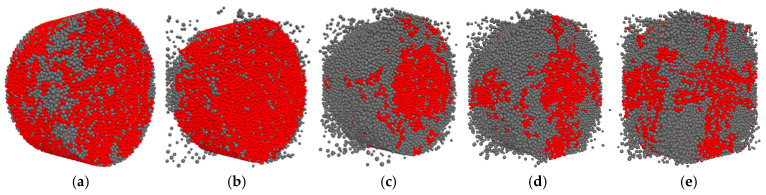
Distribution of high stress before and after loading of limonite concrete; (**a**) high stress distribution before loading; (**b**) 52.7 s^−1^; (**c**) 74.8 s^−1^; (**d**) 107.5 s^−1^; (**e**) 128.7 s^−1^.

**Figure 14:**
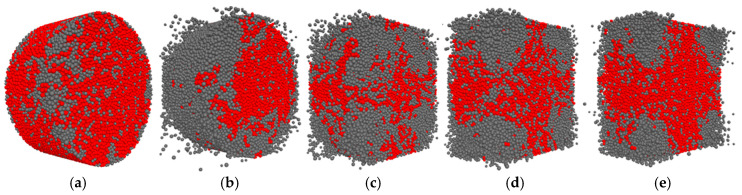
Distribution of high stress before and after loading of lead-zinc ore concrete; (**a**) high stress distribution before loading; (**b**) 19.19 s^−1^; (**c**) 41.84 s^−1^; (**d**) 53.12 s^−1^; (**e**) 65.72 s^−1^.

In this paper, not all stresses were analyzed, but only high stresses. The method of determining the high stress is to calculate the average value of all contact stresses in the specimen and then traverse all the stresses of all the contacts again (those greater than the average value are high stress contacts, and those less than the average value are low stress contacts) and calculate the angle and then count, then normalize the number of contacts, summarize them into a table, and finally plot the image. The high stress distribution before and after loading of limonite concrete and lead-zinc ore concrete is shown in Figure 13 and Figure 14. The contact anisotropy of the two types of concrete is shown in Figure 14.

Figure 13 and Figure 14 show the high stress distribution plots before and after loading limonite concrete and lead-zinc ore concrete, with the red part showing the high stress region and the gray part showing the low stress region. After comparing the high stress distribution diagrams of the two kinds of concrete before and after loading, it was found that before loading, the stress distribution of the concrete was relatively uniform, and after loading, the stress distribution of the concrete changed with the increase of strain rate, and the damaged part of the cement bond broke, showing a low stress state, and the undamaged part of the cement bond did not break, showing a high stress state, with the increase of strain rate, the number of cracks gradually increases and the percentage of high stress contact also gradually decreases, the high stress distribution graph can be corroborated with the crack distribution graph.

**Figure 15:**
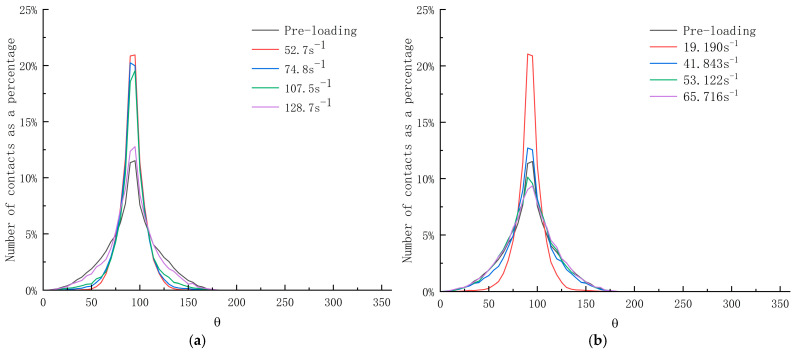
Contact anisotropy plots for limonite concrete and lead-zinc ore concrete; (**a**) limonite concrete; (**b**) lead-zinc ore concrete.

For the contact anisotropy plots of limonite concrete and lead-zinc ore concrete (Figure 15), the horizontal coordinate is the angle, ranging from 0 to 360°, and the vertical coordinate is the ratio of the number of high stress contacts to the total number of contacts. It can be found that the number of contacts at 90° (vertical direction) is the highest, i.e., the stresses in the vertical contact are more pronounced in the SHPB dynamic impact simulation.

It should be noted that the high stress distribution map, the square shape presented by the crack distribution map after high strain rates, and the high stress distribution presented by the edge particles are formed because of the presence of the domain region in the PFC6.0, where the already destroyed particles are gathered at the edge of the region, and do not have an impact on the results.

## 5. Conclusions

In this paper, based on the coupling of FDM and DEM, the SHPB impact compression test was simulated to obtain information on stress, cracks, and other parameters of concrete with different aggregates under different strain rates and analyzed by combining with the stress-strain images during the loading process, the damage state and the high stress distribution map, and obtain the following conclusions:

(1) Limonite concrete and lead-zinc ore concrete showed a significant strain rate strengthening effect, with peak stress and peak strain increasing with increasing strain rate. The stress-strain curves of the two types of concrete show a similar trend, and the relationship between DIF and strain rate is consistent with the overall existing research pattern. The DIF and dynamic compressive strength growth of lead-zinc ore concrete was greater than that of limonite concrete, and the strain rate sensitivity of lead-zinc ore concrete was stronger than that of limonite concrete.
(2) By constructing a three-dimensional numerical model, the stress-strain curve, damage pattern, and high stress distribution of concrete under dynamic loading can be in good agreement with the test results. The model reproduces the whole damage process of concrete specimens from a microscopic point of view, thus verifying the feasibility and validity of the simulation.
(3) Based on the built-in Python language module of the PFC, a program was written to carry out the parameter calibration process using unconfined uniaxial compression as the unit test. The relative errors of the compressive strength and modulus of elasticity obtained from the calibration with the test values are within 1%, which proves the effectiveness of the automatic calibration procedure for the microscopic parameters. The program greatly simplifies the calibration process of microscopic parameters and improves the computational efficiency.
(4) The degree of destruction of the specimens during the impact of the two types of concrete increased with the increase in strain rate. As the strain rate increases, the damage pattern of concrete evolves from destruction into large pieces to destruction into small pieces and then to destruction into powder. Concrete cracks first appeared at the junction of the specimen and the compression bar, and with the compaction of the specimen, cracks were generated inside the specimen, and eventually, the internal and external cracks extended and intersected with each other until destruction.

In summary, the research method provided in this paper has a good application prospect in studying the dynamic mechanical properties of concrete with different aggregates, and the coupled model established can predict the performance change of concrete well and provide some guidance for engineering. However, due to the limitation of computational ability, the established model fails to simulate the various properties of concrete specimens more completely, so the simulation results still have a certain gap compared with the experimental results. In the future, the research can be strengthened in this area, and more aspects to compare the performance differences between different aggregate concrete can be added to the study of fractal dimension so as to produce more comprehensive results.

## Figures and Tables

**Table 1:** Basic physical properties of two concrete specimens.

Performance Indicators	Specimen Type	
	Limonite	Lead-Zinc Ore
Compressive strength/MPa	42.4	44.4
Elastic modulus/GPa	33.13	36.3

**Table 2:** Macroscopic parameters of the numerical model of the specimen.

Specimen Diameter (mm)	Specimen Height (mm)	Particle Radius (mm)	Particle Density (kg/m^3^)	Initial Porosity	Initial Damping Factor
100	50	0.8~1.4	2800	0.35	0.3

**Table 3:** Parameters of rod numerical model.

Rod	Length (mm)	Diameter (mm)	Elastic Modulus (GPa)	Density (kg/m^3^)	Poisson’s Ratio
Bullet	600	100	190.3	7650	0.3
Incident rod	5000				
Transmissive rod	5000				

**Table 4:** Comparison of experimental and simulation parameters of concrete with different aggregates.

Category	Compressive Strength			Elastic Modulus		
	Experimental Value/MPa	Simulated Value/MPa	Inaccuracy/%	Experimental Value/MPa	Simulated Value /MPa	Inaccuracy/%
Limonite Concrete	42.4	42.555	0.366	33.13	33.142	0.036
Lead-Zinc Ore Concrete	44.4	44.616	0.486	36.3	36.233	0.185

**Table 5:** Particle contact microscopic parameters of different aggregate concrete fine view mechanical modeling.

Microscopic Parameters	Aggregate Types	
	Limonite	Lead-Zinc Ore
emod (Effective modulus/GPa)	0.296	0.324
kratio (Normal-Shear Stiffness Ratio)	1.5	1.5
fric (coefficient of friction)	0.1	0.1

**Table 6:** Parallel bond microscopic parameters of different aggregate concrete fine view mechanical models.

Microscopic Parameters	Aggregate Types	
	Limonite	Lead-Zinc Ore
pb_emod (Effective modulus of bond/GPa)	2.96	3.24
pb_kratio (Bond normal-shear stiffness ratio)	1.5	1.5
pb_coh (bonding cohesion/MPa)	15.6	16.5
pb_ten (bonding tensile strength/MPa)	42	44.4
pb_fa (angle of internal friction of cementation)	50	50

**Table 7:** Damage patterns of limonite concrete at different strain rates.

Category	Strain Rate			
	52.71 s^−1^	74.8 s^−1^	107.5 s^−1^	128.7 s^−1^
Experimental	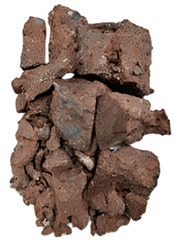	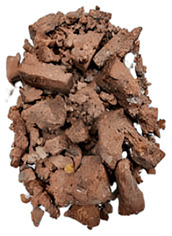	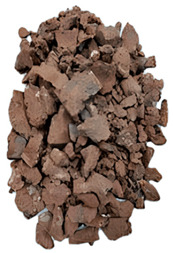	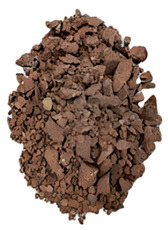
Simulation	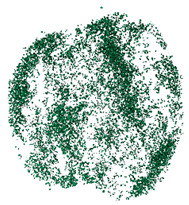	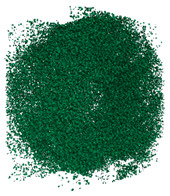	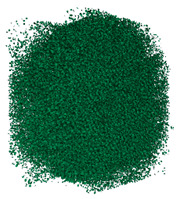	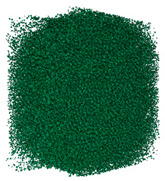

**Table 8:** Damage diagrams of lead-zinc ore concrete at different strain rates.

Category	Strain Rate			
	19.19 s^−1^	41.84 s^−1^	53.12 s^−1^	65.72 s^−1^
Experimental	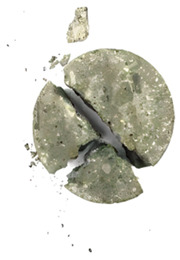	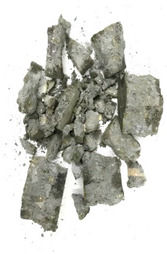	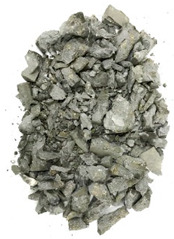	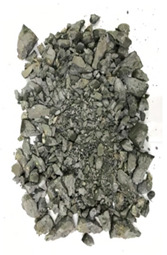
Simulation	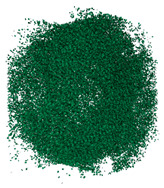	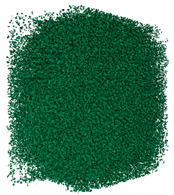	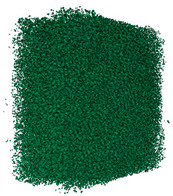	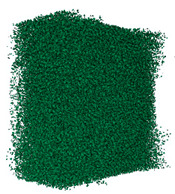

**Table 9:** Crack evolution of concrete with different aggregates for different stress scenarios at similar strain rates.

Category	20% Peak Stress	40% Peak Stress	60% Peak Stress	80% Peak Stress	Peak Stress	Damage
Limonite Concrete (strain rate-52.7 s^−1^)	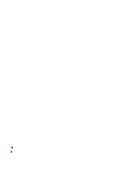	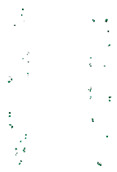	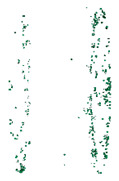	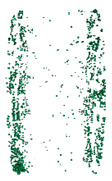	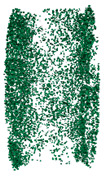	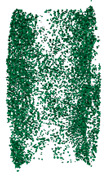
Lead-Zinc Ore Concrete (strain rate-53.12 s^−1^)	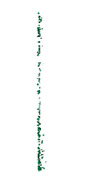	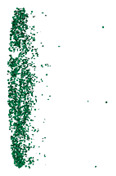	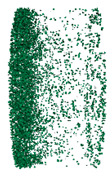	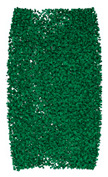	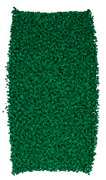	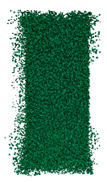

## Data Availability

Data will be made available on request.
